# Pathogenic and Therapeutic Relevance of JAK/STAT Signaling in Systemic Lupus Erythematosus: Integration of Distinct Inflammatory Pathways and the Prospect of Their Inhibition with an Oral Agent

**DOI:** 10.3390/cells8080898

**Published:** 2019-08-15

**Authors:** Alessia Alunno, Ivan Padjen, Antonis Fanouriakis, Dimitrios T. Boumpas

**Affiliations:** 1 Rheumatology Unit, Department of Medicine, University of Perugia, Ospedale S.M. della Misericordia, Edificio C, 5° piano, Piazzale Menghini 1, 06129 S. Andrea delle Fratte, Perugia, Italy; 2 Division of Clinical Immunology and Rheumatology, Department of Internal Medicine, University Hospital Center Zagreb and University of Zagreb School of Medicine, 10000 Zagreb, Croatia; 3 Rheumatology and Clinical Immunology Unit, 4th Department of Internal Medicine, “Attikon” University Hospital, 12462 Athens, Greece; 4 Department of Rheumatology, “Asklepieion” General Hospital, 16673 Athens, Greece; 5 Laboratory of Autoimmunity and Inflammation, Biomedical Research Foundation of the Academy of Athens, 11527 Athens, Greece; 6 Joint Academic Rheumatology Program, Medical School, National and Kapodestrian University of Athens, Athens, Greece and Medical School, University of Cyprus, 1678 Nicosia, Cyprus

**Keywords:** systemic lupus erythematosus, Janus kinase, protein tyrosine kinases, STAT, baricitinib

## Abstract

Four Janus kinases (JAKs) (JAK1, JAK2, JAK3, TYK2) and seven signal transducers and activators of transcription (STATs) (STAT1, STAT2, STAT3, STAT4, STAT5A, STAT5B, STAT6) mediate the signal transduction of more than 50 cytokines and growth factors in many different cell types. Located intracellularly and downstream of cytokine receptors, JAKs integrate and balance the actions of various signaling pathways. With distinct panels of STAT-sensitive genes in different tissues, this highly heterogeneous system has broad in vivo functions playing a crucial role in the immune system. Thus, the JAK/STAT pathway is critical for resisting infection, maintaining immune tolerance, and enforcing barrier functions and immune surveillance against cancer. Breakdowns of this system and/or increased signal transduction may lead to autoimmunity and other diseases. Accordingly, the recent development and approval of the first small synthetic molecules targeting JAK molecules have opened new therapeutic avenues of potentially broad therapeutic relevance. Extensive data are now available regarding the JAK/STAT pathway in rheumatoid arthritis. Dysregulation of the cytokines is also a hallmark of systemic lupus erythematosus (SLE), and targeting the JAK/STAT proteins allows simultaneous suppression of multiple cytokines. Evidence from in vitro studies and animal models supports a pivotal role also in the pathogenesis of cutaneous lupus and SLE. This has important therapeutic implications, given the current paucity of targeted therapies especially in the latter. Herein, we summarize the currently available literature in experimental SLE, which has led to the recent promising Phase II clinical trial of a JAK inhibitor.

## 1. JAK/STAT Pathway and Autoimmunity: An Overview

### 1.1. Physiology and Pathophysiology

Janus kinases (JAKs) are intracellular protein tyrosine kinases (PTK); to date, four have been identified in mammals: JAK1–3 and TYK2. JAK1, JAK2, and TYK2 are ubiquitously expressed, whereas JAK3 is expressed only by cells of hematopoietic origin [1]. JAKs contain different domains, namely, an N-terminal FERM-domain, an SH2-like domain, a pseudokinase domain (JAK homology 2, JH2), and the catalytically active, signaling PTK domain (JH1). The FERM and SH2-like domains mediate the interaction of JAKs with their receptors and regulate their kinase activity [2,3].

The seven mammalian signal transducer and activator of transcription (STAT) proteins (STAT1–4, STAT5A, STAT5B, and STAT6) are classic transcription factors able to modulate gene transcription via the engagement of DNA regulatory elements (DREs) [4]. The JAK/STAT signal transduction pathway is responsible for mediating signals of over 50 type I and type II cytokines, growth factors, and hormones [5,6] (Table 1).

Binding of JAK to its receptor leads to conformational changes in the cytoplasmic portion of the latter, initiating activation of JAKs associated to the receptor. Subsequently, JAKs mediate phosphorylation at the specific receptor tyrosine residues, which then serve as docking sites for STATs and other signaling molecules. The notion that unphosphorylated STATs are present in the cytoplasm as free monomers has been recently challenged by the demonstration that they exist as dimers and higher molecular mass complexes [7,8]. Once recruited to the receptor, STATs also become phosphorylated by JAKs, on a single tyrosine or serine residue. This leads to conformational change of the preformed dimer, translocation to the nucleus, and binding to members of the gamma-activated site (GAS) family of enhancers. Of note, all STAT3 constitutively traffic from the cytoplasm to the nucleus and back, regardless of their phosphorylation status [9]. However, phosphorylated STAT3 has increased residence time in the nucleus, due to DNA binding. Furthermore, not all phosphorylated STAT3 is nuclear and all STATs, including those unphosphorylated, can be transcriptionally active, albeit on different sets of genes [8,10].

The JAK/STAT signaling pathway is tightly regulated on multiple levels, allowing a fine-tuning between prompt activation and timely signal termination. For the latter, JAK dephosphorylation, the production of inhibitory proteins such as the suppressor of cytokine signaling (SOCS) family, or the autoinhibitory mechanism of JH2–JH1 domain interaction are some of the operant mechanisms to prevent aberrant activation of the pathway. Accordingly, any condition affecting one of these regulatory checkpoints (modulating JAK and STAT function or leading to cytokine overexpression) leads to loss of control and potentially to disease [11].

Over the last three decades, studies have addressed the involvement of the JAK/STAT pathway in disease, particularly inflammation and cancer. It is considered a key player in tumorigenesis, as JAK mutations (e.g., rearrangements in multiple fusion gene partners, amplifications or even epigenetic changes, leading to aberrant activation) have been identified in hematologic malignancies, such as acute lymphoblastic leukemia and lymphomas [12], but also in solid neoplasms like triple-negative breast cancer [13].

### 1.2. JAK/STAT System: Balancing between Autoimmunity and Immune Deficiency

For immune-mediated diseases, both activating and inactivating mutations of JAKs and STATs may cause abnormalities in immune homeostasis (Table 2).

Loss-of-function mutations of JAK3 are associated with severe combined immune deficiency (SCID) [14]; similarly, loss-of-function mutations of STAT1 and STAT2 were also found responsible for immunodeficiency [15], whereas STAT5 deficiency can lead to both immunodeficiency and autoimmunity. Gain-of-function mutations of STAT3 cause early-onset lymphoproliferative disease and autoimmunity [16], those of STAT1 lead to chronic mucocutaneous candidiasis, but also autoimmunity [17], and dominant negative mutations of STAT3 cause a hyper-IgE syndrome, known as Job syndrome [18]. Finally, polymorphisms of STAT genes have been linked to susceptibility for systemic autoimmune diseases, including rheumatoid arthritis (RA) and systemic lupus erythematosus (SLE) [19,20]. Abnormal expression of JAK/STAT modulators is another cornerstone in the development of autoimmunity. The protein inhibitor of activated STATs (PIAS) family member 2 encodes a transcriptional co-regulator in the STAT and p53 pathways and is downregulated in SLE [21]. Likewise, SOCS1 and SOCS3, two highly specific negative regulators of JAKs, are defective in SLE [22,23].

## 2. The JAK/STAT Pathway in Rheumatic Diseases

The term autoimmune rheumatic diseases (AIRDs) encompasses a wide range of conditions that commonly affect the joints, but can virtually target any organ system, and are caused by aberrations of the immune system. The robust evidence on the critical role of the JAK/STAT pathway in type I and type II cytokine signaling, together with the clinical efficacy of its blockade in hematologic malignancies, prompted research also in the field of rheumatic diseases. After early studies demonstrating that tofacitinib, a non-selective inhibitor of JAK1, 2, and 3, prevents transplant rejection in mice and non-human primates [24], pivotal studies exploring JAK/STAT blockade in experimental arthritis paved the way for rheumatic diseases [25]. To this end, several models of arthritis were explored with tofacitinib reducing disease severity, if administrated at the onset of murine collagen-induced arthritis (CIA), rat adjuvant-induced arthritis, and K/BxN serum transfer arthritis. Importantly, amelioration of CIA could be achieved in established disease. Tofacitinib effectively reduced inflammation and joint damage also in SCID mice implanted with synovium and cartilage from patients with RA [26], through the inhibition of interferon (IFN)-γ, interleukin (IL)-6, IL-8, and IL-17. Baricitinib, an oral selective inhibitor of JAK1 and JAK2 [27], also reduced the severity of CIA and rat adjuvant-induced arthritis. Suppression of IFN-γ and subsequent migration stimuli of fibroblast-like synoviocytes, as well as blockade of osteoclast formation, have been reported as mechanisms underlying the effect of baricitinib [28]. Selective inhibitors of JAK/STAT pathways, such as decernotinib (JAK3) and filgotinib (JAK1 and JAK2), demonstrated efficacy in ameliorating CIA [29,30]. Following these preclinical data, several clinical trials tested these compounds in human RA; tofacitinib and baricitinib are already approved for its treatment, while others are still under investigation. In parallel, inhibitors of the JAK/STAT pathways have also been studied in other rheumatic diseases [31,32,33]. Among AIRDs, systemic lupus erythematosus (SLE) represents the prototype of systemic autoimmune disorders [34]. Despite increasing knowledge about its pathogenesis, the heterogeneity of the disease along with the unsatisfactory and sometimes absent response to conventional treatment represent challenges for rheumatologists in routine practice. Given the multiple failed trials of biologic agents over the last decade (for a variety of reasons that go beyond the scope of this review [35]), the identification of novel therapeutic targets and ultimately the development of novel therapeutic strategies are a top priority in the research agenda for SLE [36].

## 3. The JAK/STAT Pathway in Experimental SLE

The rationale for the first experiments investigating the role of the JAK/STAT pathway in the pathogenesis of SLE was based on two premises: first, interferons (IFN) type I (α/β) and II (γ) are well-established and important players in SLE pathophysiology; second, the JAK/STAT cascade had been identified responsible for the signal transduction from the activated IFN receptor to the nucleus [37,38].

### 3.1. Evidence from In Vitro Studies

In their study of gene expression in CD3+ T cells of 12 patients with SLE, Kawasaki et al. demonstrated that the expression of IFN regulated factor (IRF)-related genes was upregulated in patients with active disease. The pathway network analysis performed in this study suggested that the JAK/STAT pathway may regulate IRF-related genes [39]. More recently, in a study on peripheral blood mononuclear cells (PBMCs) from 52 SLE patients, T cells from patients carrying the STAT4 risk allele rs7574865[T] had an augmented STAT4 phosphorylation response to IFN-α and IL-12, leading in turn to a pronounced IL-12-induced production of IFN-γ in both CD4+ and CD8+ cells. TYK2 blockade inhibited the IL-12 and IFN-α activation of T cells, while JAK2 blockade suppressed cellular activation induced by IFN-γ [40]. In a study on antibody-secreting cells (ASCs) (CD20-D19^low^-D38^high^) from blood samples of 57 patients with SLE, anti-dsDNA- and anti-ENA-specific ASCs were shown to express receptors for plasma-cell niche cytokines (such as IL-6, IL21, CXCL2, BAFF, and APRIL). These cytokines, in turn, were shown to promote IgG and autoantibody production in a STAT3-dependent manner. These effects were suppressed by the use of Stattic and ruxolitinib [41].

### 3.2. Evidence from In Vivo Studies

Most in vivo experimental data on the role of the JAK/STAT pathway focus on three aspects of the disease—lupus nephritis (LN), cutaneous lupus, and serological parameters. Potential therapeutic implications of JAK and/or STAT inhibition recognized in murine models of lupus are presented in Table 2, on a study-by-study basis. More detailed findings of these studies are outlined below, divided in particular domains of the disease (Table 3).

#### 3.2.1. Lupus Nephritis

In their study performed on the Murphy Roths Large/lymphoproliferation (MRL/lpr) murine model of SLE, Dong et al. demonstrated that expression and activation of STAT1 were upregulated in the kidney, mostly in glomerular mesangial cells. In a second part of their study, using a culture of mesangial cells, they showed an inhibitory effect of a JAK2-STAT1 inhibitor (AG-490) on STAT1 activation induced by IFN (γ and α) [38]. Increased glomerular and tubular expression of STAT1 was also found in renal biopsy specimens obtained from patients with diffuse proliferative LN. STAT1 expression correlated with overall disease activity, as well as with serum creatinine levels and worse renal outcome [51]. The first in vivo study of therapeutic exploitation of the JAK/STAT pathway in lupus was performed by Wang et al., who administered the aforementioned JAK2-STAT1-inhibiting compound AG-490 to MRL/lpr mice with LN. Compared to vehicle-treated mice, administration of AG-490 resulted in less pronounced inflammation (glomerulonephritis, interstitial nephritis, vasculitis, and even sialadenitis as an extra-renal feature), as well as suppression of chemokines, IFN-γ, and major histocompatibility complex class II (MHC-II) molecules on the surface of renal cells. Treatment with AG-490 also led to lower levels of blood urea nitrogen, serum creatinine, and proteinuria, and to decreased IgG and C3 deposition in glomerular cells. Accordingly, immunohistochemistry revealed lower expression of STAT1 in glomerular, tubular, and interstitial cells [42]. The effect of a selective JAK2 inhibitor (CEP-33779) on LN mice was evaluated in a pivotal study by Lu and coworkers, who showed that CEP-33779 can both protect MRL/lpr mice from developing renal involvement (preventive study model) and also ameliorate already established disease in New Zealand black/New Zealand white F1 (NZB/WF1) mice (established disease model). In the first model, treatment (compared to vehicle-treated mice) led to alleviation of splenomegaly, lymphadenopathy, glomerulonephritis, and tubulointerstitial nephritis, accompanied by a decline in phosphorylated STAT3 (pSTAT3). In established disease, CEP-33779 led to increased survival, decline in proteinuria, resolution of histological features of renal disease, and decrease in pSTAT3. Intriguingly, CEP-33779 lowered the levels of long-lived plasma cells in the spleen (at all doses) and even in the bone marrow (at the highest dose). This effect may have therapeutic implications in human LN, given that long-lived plasma cells are responsible for the production of antibodies and thus in the perpetuation of the autoimmune response in long-standing disease. Conversely, treatment with CEP-33779 did not affect levels of splenic short-lived plasma cells, which may be associated with fewer immunosuppression-related side effects (i.e., infections) and potentially better responses to vaccines [43,52].

In a study of human glomerulonephritides by Arakawa et al., increased glomerular staining of STAT3 was observed in renal biopsies of patients with LN. In patients with different types of glomerulonephritides, STAT3 activation highly correlated with glomerular and tubulointerstitial cell proliferation, interstitial fibrosis, and the level of renal injury. Moreover, downregulation of STAT3 was observed following glucocorticoid (GC) treatment [53]. However, these results should be interpreted with caution in regard to SLE, since only 9/45 patients had LN and no separate analysis was performed in this subgroup.

In a study on the NZB/WF1 murine model of SLE, Ripoll et al. examined the effect of tofacitinib on renal lesions, comparing it against cyclophosphamide (CYC) and mycophenolate mofetil (MMF). Mice with established nephritis treated with tofacitinib had higher cumulative survival and a more pronounced reduction of proteinuria compared to vehicle-treated animals, a reduction comparable to the one observed in mice treated with CYC and MMF. Tofacitinib-treated mice demonstrated milder glomerular, tubular, and interstitial lesions, as well as fewer renal deposits of IgG and C3, compared to controls. Numbers of both T cells and macrophages were lower in tofacitinib-treated mice, as well as the expression of STAT-regulated genes and several inflammatory mediators. Treatment with tofacitinib led to significantly lower secretion of TNF-α, IFN-α, and IL-17. Since the latter has been linked to renal injury associated with immune-complex deposition, the authors postulated that inhibition of IL-17 through JAK3-mediated inhibition of STAT3 might have therapeutic implications in LN [44].

Similar effects of tofacitinib were observed in another study on NZB/WF1 mice. Compared to vehicle-treated mice, tofacitinib-treated mice (with and without dexamethasone) exhibited lower levels of proteinuria, decreased frequency of severe glomerulonephritis, lower glomerulus scores, less pronounced interstitial nephritis, and lower intensity of renal IgG and C1q deposition. Less pronounced splenomegaly was also noted. Similar findings were observed in MRL mice treated with tofacitinib and dexamethasone in the same study, indicating that the effect of treatment may be independent of genetic background. Expression of several cytokines (IL-6, Il-2, IFN-α) and of two IFN-signaling pathway genes, *Ifit3* and *lsg15*, was reduced in tofacitinib-treated animals [45].

In a study exploring the potential of tofacitinib to prevent renal disease in MRL/lpr mice, Furumoto et al. observed less pronounced histopathologic features of LN, as well as decreased immune complex deposition in mice treated with tofacitinib at 10 weeks of age, accompanied by a lower level of proteinuria. A significant decline in proteinuria, serum creatinine levels, and blood urea nitrogen was also observed in the model of established disease in fourteen-week-old mice [46]. Expression of several IFN-signaling genes (type I IFN genes, *Mx1*, *Stat1*, *Isg15*, *Ifit1*) was decreased in tofacitinib-treated mice in both the preventive and established disease model [46].

Another study showed the efficacy of CDDO-Me (C-28 methyl ester of 2-cyano-3,12-dioxoolean-1,9-dien-28-oic acid), a compound targeting JAK1 and STAT3, in ameliorating renal disease, both in a preventive setting (on two mouse models of LN, B6-Sle1.Sle3 and MRL/lpr) and an established setting (mouse model NZM2410) [47]. The role of STAT3 in LN pathogenesis is also supported by a study by Ding and colleagues, wherein B6.MRL/lpr B-cell STAT3 knockout mice had a markedly reduced renal inflammatory infiltrate, as well as less pronounced renal IgG and C3 deposition, compared to controls [54]. Moreover, two additional studies assessed the role of selective STAT3 inhibition in LN, through the administration of the compound S31-201 (Stattic) to MRL/lpr mice. In the first study, mice treated with Stattic had a lesser degree of glomerulonephritis, less renal IgG deposition, as well as delayed onset of proteinuria [48]. Du and coworkers, on the other hand, focused on tubulointerstitial renal lesions, showing that this STAT3-selective inhibitor ameliorated tubule injury, inflammation, and interstitial fibrosis. In addition to STAT3 inhibition, Stattic led to increased phosphorylation (i.e., activation) of STAT1; this could indicate that STAT3-mediated signaling may be more important than STAT1 for LN pathogenesis (since renal disease was ameliorated in spite of increased STAT1 phosphorylation) [49].

#### 3.2.2. Cutaneous Lupus

Despite the fact that skin lesions are included in the phenotype of MRL/lpr mice (erythematous scarring rash with alopecia) [55], efficacy of JAK/STAT inhibition on skin was not reported in the majority of the above described studies. An exception was the study by Furumoto et al., where tofacitinib ameliorated both clinical and histological features of lupus-associated skin inflammation in MRL/lpr mice [46].

Another study in the same animal model examined the effect of ruxolitinib, a relatively specific JAK1 and JAK2 inhibitor. The latter attenuated the development of lupus-associated skin lesions, reduced the inflammatory infiltrate (as well as T cells in the infiltrate), and epidermal hyperplasia, downregulating the expression of IFN response genes. Interestingly, the drug exerted no effect on other lupus features. However, this potential lack of systemic effect does not diminish potential therapeutic implications of ruxolitinib in cutaneous lupus, given that, being a small molecule, the drug may be formulated for topical use [50]. Interestingly, ruloxitinib was efficacious in a primary myelofibrosis patient who was treated due to chilblain lupus [56]. Pronounced expression of activated JAK2 was seen in the aforementioned patient, but also in several other patients with discoid and subacute cutaneous lupus (in contrast to patients with psoriasis and atopic dermatitis), reported by the same group of investigators [57]. Interestingly, the use of baricitinib was reported to reverse alopecia areata in a patient without other features suggestive of lupus [58].

#### 3.2.3. Serology and Autoantibodies

In the study by Wang et al., application of the JAK2-STAT1 inhibitor AG-490 led to a decline in serum anti-dsDNA antibody levels [42]. Administration of an even more selective JAK2-targeting compound led to a decrease in the titer of antinuclear antibodies, anti-dsDNA, and anti-Smith antibody levels, both in a preventive model (MRL/lpr mice) and a model of established LN (NZB/WF1 mice) [43]. A decrease in anti-dsDNA levels was also observed in the aforementioned tofacitinib study on NZB/WF1 mice, with the decrease in tofacitinib-treated mice being more profound compared to mice treated with CYC and MMF [44]. A fall in anti-dsDNA antibodies was also observed in CDDO-Me-treated mice [47], as well as tofacitinib-treated NZB/WF1 [36] and MRL/lpr mice (the effect was observed both in the preventive and in the established disease models) [37]. In the latter case, the decline in the titer of antinuclear antibodies reached statistical significance only in the preventive study setting [46]. A similar finding was observed in the study by Edwards et al., who observed delayed development of anti-dsDNA IgG antibodies (and their lower serum titers) only in mice treated with a long-term regimen of Stattic (a STAT3 inhibitor), and not in mice treated with a short-term regimen [48]. This effect of STAT3 inhibition is in accordance with significantly reduced levels of antinuclear, anti-dsDNA. and anti-snRNP in sera of eight-month-old B6.MRL/lpr B-cell STAT3 knockout mice, when compared to controls [54].

Higher serum levels of C3 observed in mice receiving the long-term regimen of Stattic [39] are in line with findings of decreased C3 and immune complex deposition in kidneys treated with inhibitors of the JAK/STAT pathways [42,44,45]. Higher levels of C3 were also observed in lupus-prone MRL/lpr mice treated with a selective JAK2 inhibitor [43].

Interestingly, STAT signaling may be important for the association (or even “coupling”) between the serological profile and the histologic type of LN. In a study on NZM2328 mice, STAT4-deficient mice developed class IV lupus nephritis with lower levels of anti-dsDNA antibodies, whereas STAT6-deficient mice developed much less severe type II lupus nephritis, the onset of which was significantly delayed in comparison to wild type mice. In contrast to their STAT4-deficient counterparts, STAT6-deficient mice developed high antibody levels [59].

#### 3.2.4. Lupus-Associated Vascular Dysfunction

Inhibition of the JAK/STAT signaling pathway could also ameliorate lupus-associated vascular dysfunction. In the study by Furumoto et al., application of tofacitinib in MRL/lpr mice was associated with improvement in endothelium-dependent vasorelaxation, a tendency towards increased capacity of endothelial progenitor cells to differentiate into mature endotheliocytes, as well as increased HDL cholesterol levels [46]. The relevance of these findings to the pathogenesis of lupus-associated atherosclerosis is limited by the short-term application of the drug in this study.

To summarize all available experimental evidence, it is encouraging that similar therapeutic effects were observed using different molecular targets within the JAK/STAT system, in vitro as well as in vivo, in several murine models differing in genetics, clinical presentation, disease kinetics, and features of kidney histopathology [52,55]. More importantly, observations from preventive study models were replicated in models of established disease (Table 3). Nevertheless, results observed in the experimental setting of animal models should always be interpreted with caution. Selection of the most appropriate molecular target within the JAK/STAT signaling pathway still remains an open question, especially since the roles of each of the tested molecules in animal models might not be identical in humans.

## 4. From Bench to Bedside: Data from Clinical Trials and Future Perspectives

Based on the previous experimental and preclinical data, baricitinib, an oral selective JAK1 and JAK2 inhibitor approved for the treatment of rheumatoid arthritis, was recently tested in a multicenter randomized, placebo-controlled, 24-week phase II study, in 314 patients with extrarenal SLE, involving mainly skin and joints. Patients were randomized 1:1:1 to receive two doses of baricitinib (4 mg and 2 mg/day, respectively) or placebo, on top of background therapy [60]. The primary endpoint of the study was the proportion of patients achieving resolution of arthritis or rash (defined by SLEDAI-2K) at week 24. Mean SLEDAI-2K at baseline was ≈ 9, with a mean cutaneous lupus erythematosus disease area and severity index (CLASI) score of 3.8–4.9 and a swollen joint count (SJC) of 5.2–5.5, overall indicating a disease of moderate severity. Importantly, more than 70% of patients were on glucocorticoids (GCs), with almost half receiving a prednisone equivalent dose of ≥7.5 mg/day.

The low dose of 2 mg/day did not confer any additional benefit over placebo, either for the primary or any of the secondary endpoints. However, more patients in the 4 mg/day baricitinib group met the primary endpoint (67 vs. 53% with placebo, respectively, OR 1.8), in a result that marginally met statistical significance (*p* = 0.041). Baricitinib 4 mg/day was also associated with a two-fold higher possibility of attaining the composite response index, SLE responder index-4 (OR 2.0, *p* = 0.01) and a lower risk of flares (hazard ratio 0.6, *p* = 0.020). There was no difference in the CLASI score and the number of SJC. Even though the tender joint count (TJC) was different between the two groups, this finding may be subject to patient bias, especially in the context of no difference in SJC, a feature also subject to patient bias [61]. Regarding safety, overall no important safety signals were raised, although infections were more common in both baricitinib arms.

Several comments can be made based on these results. First, a treatment difference of 14% may seem moderate at best; however, to put things into perspective, a difference of this magnitude (i.e., ≈10–15%) led to the approval of belimumab following the successful BLISS trials (of course, in a vastly larger patient population). More problematic is the finding of no significant response in swollen joints and, most importantly, skin disease, a finding discordant from the data in animal models [52]; however, this may well be attributed to the relatively low baseline CLASI score in both arms, which may be inadequate for baricitinib to demonstrate a meaningful difference. Finally, the handling of GCs during the study was unusual, as tapering was not allowed between weeks 16 and 24, and the authors provide no data on the proportion of patients that successfully tapered or even discontinued GCs; high background therapy, especially GCs, can account for the lack of effect of drugs in the SLE trials.

Despite these limitations, the successful phase II study of baricitinib justifies further study of the drug in lupus and two multicenter phase III randomized placebo-controlled trials are currently underway in extrarenal disease (NCT03616912, NCT03616964, both recruiting participants). The track history of the multiple failed trials of new drugs highlights the challenge of clinical trial design in SLE [62]. In essence, the expected moderate differences conferred by drugs under study typically require large numbers of patients to reach statistical significance. Accordingly, the disproportionately low number of referral centers often necessitates the inclusion of trial sites with less experience in lupus and the complex indices used in lupus trials.

## 5. Conclusions and Perspectives

Targeting of the JAK/STAT pathway represents a milestone in the treatment of autoimmune diseases; different inhibitors are currently being tested under various conditions. Among the latter, SLE is probably the most challenging, as it has become notorious for the multiple failed phase III clinical trials of biologic agents. Several lines of evidence suggest that, given the complexity of the disease, multiple pathways may need to be targeted to ensure effectiveness. To this end, because of the reported dysregulation of multiple cytokines (a key feature of SLE), the prospect of the simultaneous inhibition of multiple cytokines with an oral formulation with the potential to be less expensive is attractive. The initial evidence of effectiveness of the recent phase II trial of baricitinib dictates cautious optimism, while awaiting a larger trial. We remain optimistic that the wealth of experimental data regarding the role of the JAK/STAT pathway in SLE will be rewarded by the approval of a new drug for this demanding disease.

## Figures and Tables

**Table 1:** Member proteins of the JAK/STAT system and upstream molecules, which signal via the respective protein.

JAK/STAT Member	Proteins Signaling through Each JAK/STAT Member	Proposed Effect of Therapeutic Inhibition (Simplified)
JAK1	Interferons (α, β, γ), IL-2, IL-4, IL-6 family cytokines, IL-7, IL-9, IL-10 family cytokines	Immunosuppression
JAK2	EPO, TPO, GM-CSF, G-CSF, IL-3, IL-5, interferon-γ, GH, leptin	Immunosuppression, inhibition of hematopoietic cell differentiation
JAK3	IL-2, IL-4, IL-7, IL-9, IL-15, IL-21	Immunosuppression
TYK2	Interferons (α, β, γ), IL-12, IL-23	Immunosuppression
STAT1	Interferons (α, β, γ)	Immunosuppression
STAT2	Interferons α and β	Immunosuppression
STAT3	IL-6 family cytokines, IL-10 family cytokines, G-CSF, leptin, IL-21, IL-27, several oncogenes and growth factors	Immunosuppression, inhibition of hematopoietic cell differentiation
STAT4	Interferons α and β, IL-12, IL-23	Immunosuppression, inhibition of Th1 cell differentiation
STAT5	GM-CSF, GH, TPO, EPO, IL-2, IL-3, IL-5, IL-7, IL-9, IL-15	Immunosuppression, inhibition of hematopoietic cell differentiation
STAT6	IL-4, IL-13	Immunosuppression, inhibition of Th2 cell differentiation

JAK, Janus kinase; TYK, tyrosine kinase; STAT, signal transducer and activator of transcription; IL, interleukin; EPO, erythropoietin; TPO, thrombopoietin; GM-CSF, granulocyte-macrophage colony-stimulating factor; G-CSF, granulocyte colony-stimulating factor; GH, growth hormone.

**Table 2:** Mutations of JAKs and STATs leading to a pathologic phenotype.

Target	Type of Mutation	Phenotype	Ref
JAK3	Loss-of-function	Severe combined immune deficiency (SCID)	[14]
STAT1 and STAT2	Loss-of-function	Immunodeficiency	[15]
STAT5	Loss-of-function	Immunodeficiency or autoimmunity	
STAT3	Gain-of-function	Early-onset lymphoproliferative disease and autoimmunity	[16]
STAT1	Gain-of-function	Chronic mucocutaneous candidiasis and autoimmunity	[17]
STAT3	Dominant negative mutations	Hyper-IgE syndrome	[18]
Different STATs	Polymorphisms	Susceptibility for systemic autoimmune diseases	[19,20]

**Table 3:** In vivo studies on murine models of lupus, assessing the potential therapeutic role of JAK/STAT inhibition.

Study [ref.]	Compound	Main Molecular Target	Mouse Model	Design	Main Effects
Wang et al. [42]	AG-490	JAK2	MRL/lpr	P	↓ nephritis, ↓ sialadenitis, ↓ serum dsDNA antibodies
Lu et al. [43]	CEP-33779	JAK2	MRL/lpr, BWF1	P (MRL/lpr), E (BWF1)	P: ↓ nephritis, ↓ splenomegaly, ↓ lymphadenopathy, ↑ serum C3 levels; E: ↓ nephritis, ↑ survival, ↓ level of long-living plasma cells in the spleen and bone marrow; Both (serum): ↓ ANA, ↓ anti-dsDNA and ↓ anti-Smith antibodies
Ripoll et al. [44]	Tofacitinib	JAK3 and JAK1	NZB/NZWF1	E	↓ nephritis, ↑ survival, ↓ serum anti-dsDNA antibodies
Ikeda et al. [45]	Tofacitinib	JAK3 and JAK1	NZB/NZWF1, MRL	P	↓ nephritis, ↓ serum anti-dsDNA antibodies
Furumoto et al. [46]	Tofacitinib	JAK3 and JAK1	MRL/lpr	P, E	↓ nephritis, ↓ skin lesions, ↓ serum anti-dsDNA antibodies, ↓ ANA, ↓ vascular dysfunction
Wu et al. [47]	CDDO-Me	JAK1 and STAT3	B6-Sle1.Sle3, MRL/lpr, NZM2410	P (B6-Sle1.Sle3, MRL/lpr), E (NZM2410)	↓ nephritis, ↓ serum anti-dsDNA antibodies
Edwards et al. [48]	Stattic	STAT3	MRL/lpr	P	↓ nephritis, ↓ serum anti-dsDNA antibodies, ↑ serum C3 levels
Du et al. [49]	Stattic	STAT3	MRL/lpr	P	↓ renal tubulointerstitial lesions
Chan et al. [50]	Ruxolitinib	JAK1 and JAK2	MRL/lpr	E	↓ skin lesions

P, preventive; E, established disease; ↑ increase; ↓ decrease; MRL/lpr, Murphy Roths Large/lymphoproliferation; MRL, Murphy Roths Large; NZB/NZWF1, New Zealand black/New Zealand white F1; BWF1, black white F1 (the same as NZB/NZWF1); NZM2410, New Zealand mixed 2410.
